# A Comparative Bioinformatic Analysis of Optic Nerve Axon Regeneration Lipidomes Using the *Xenopus laevis* as a Model System

**DOI:** 10.3390/mps8050110

**Published:** 2025-09-15

**Authors:** Vernon S. Volante, Fiona L. Watson, Sanjoy K. Bhattacharya

**Affiliations:** 1 Miami Integrative Metabolomics Research Center, Bascom Palmer Eye Institute, University of Miami, Miami, FL 33136, USA; vsv24@miami.edu; 2 Department of Ophthalmology, Morsani College of Medicine, University of South Florida, Tampa, FL 33602, USA; 3 Department of Biology, Washington and Lee University, Lexington, VA 24450, USA; watsonf@wlu.edu

**Keywords:** lipidomics, MetaboAnalyst, LipidOne, metabolomics workbench, statistical analysis, bioinformatics

## Abstract

Lipidomics is a rapidly growing branch of metabolomics that identifies lipid compositions of samples to learn more about disease and identify potential novel therapeutic targets. In the context of ophthalmology, lipidomic research has increased our understanding of optic nerve regeneration. The diversity of experimental designs for lipidomic research and the large datasets generated are two obstacles that must be addressed by bioinformatic tools to perform statistical analysis on lipidomics data. Our study provides an objective comparison of the features in two freely accessible web-based bioinformatics tools, MetaboAnalyst 6.0 and LipidOne 2.3, for analyzing an optic nerve regeneration model lipidome. A publicly available lipidomic dataset of the optic nerve axon regeneration model, *Xenopus laevis*, was used to compare the analytic capabilities of both tools. Though both tools offered univariate and multivariate analysis methods, MetaboAnalyst 6.0 had advantages in customizable data processing, normalization, analysis, and image generation. It also offered consistent multiple-comparison testing correction and comprehensive results/dataset export. Meanwhile LipidOne 2.3 uniquely allowed for univariate and multivariate analysis of lipid classes and lipid building blocks.

## 1. Introduction

Lipids play essential roles in biological systems as constituents of cellular membranes and lipid particles, cellular signaling, transport, protein trafficking, growth, differentiation, and energy storage [1]. With lipids having such a robust role in systems biology, identification and quantification of lipid species within physiological and pathological samples can elucidate disease-specific lipid biomarkers, as well as identify novel therapeutic targets by investigating the role of specific lipid species and metabolic pathways in disease mechanisms [2]. Lipidomics is a rapidly growing branch of metabolomics that allows researchers to comprehensively study the complete lipid profile within a cell, tissue, or organism [3].

It has been well studied that lipids play an important role in membrane expansion during axon regeneration, as lipids are inserted into the axonal membrane via precursor vesicles [4,5]. Therefore, in ophthalmology, lipidomic analysis is an avenue to understand more about the mechanisms of axon regeneration and identify potential targets for therapeutic induction and enhancement of optic nerve regeneration to treat pathologies such as glaucoma and optic neuropathy. Lipidomics has revealed alterations in lysoglycerophospholipids and phosphatidylcholine (PC)-related species in mammalian optic nerve regeneration models [5], promotion of central nervous system (CNS) axon regeneration through the inhibition of cholesterol synthesis [6], and enhancement of regeneration by directing lipid metabolism toward phospholipid (PL) synthesis rather than triglyceride storage [7].

Lipidomic analysis follows a typical workflow that involves sample collection and preparation, data acquisition, data processing (including identification of lipid species and statistical analysis), and data interpretation [8]. A commonly used technique to analyze thousands of lipids in a biological sample is through liquid chromatography coupled with mass spectrometry (LC/MS), followed by identification and quantification of lipid molecular species through tools such as LipidSearch and MS-DIAL [9,10]. Bioinformatic tools are then used for statistical and pathway analyses, many of which are available as web-based platforms. Statistical analysis tools must consider that lipidomic research is approached with varying experimental aims, and that such studies analyze large datasets with hundreds to thousands of lipids [11]. To address the diversity of experimental aims, many web-based tools offer univariate analysis techniques for two-group and multi-group comparisons such as fold change analysis, *t*-test, volcano plot, one-way Analysis of Variance (ANOVA), and correlation analysis, as well as multivariate analysis options such as principal component analysis (PCA) and partial least squares discriminant analysis (PLS-DA). The issue that arises from lipidomics’ large datasets is the increased chance of finding false positives [11]. Therefore, correction for multiple comparison testing, the most common being the Benjamini–Hochberg procedure (or false discovery rate, FDR), is required in lipidomic analysis and is often an available option within bioinformatic tools.

One freely accessible web-based bioinformatics tool for lipidomic statistical analysis is MetaboAnalyst [12,13]. The platform was first released in 2009 as a means to simplify metabolomic data analysis and improve user accessibility [14]. Its first iteration served as a pipeline for metabolomics analysis; it was capable of processing, normalizing, and providing statistical analysis methods for various metabolomic data such as compound concentration tables, spectrally binned data, nuclear magnetic resonance spectroscopy (NMR) or MS peak lists, and gas chromatography (GC) or LC/MS spectra [14]. Since then, MetaboAnalyst has undergone multiple updates to upgrade its functions and add new features. Versions 1.0–3.0 improved statistical and functional analysis capabilities of targeted metabolomics data, while subsequent versions increased support for untargeted metabolomics data from LC-MS experiments [15]. Two modules are available for statistical analysis: one for single-factor analysis and one that utilizes a metadata table to consider other factors/covariates. Prior to analysis, MetaboAnalyst processes the data through integrity checking, filtration, and normalization [12]. Integrity checking involves replacement of missing values (automatically with half of the minimum positive value in the data, or user-specified using other missing value estimation methods) and identification and removal of outliers [16]. After integrity check, data filtration is performed to reduce the number of hypothesis tests and increase power, making this step strongly recommended for untargeted lipidomics datasets due to the large number of variables present [17]. Filtration can remove variables with low repeatability, variables that are near constant throughout experiment conditions, and variables with very small values. Finally, normalization is performed to transform the dataset into a more ‘bell curve’ distribution which is assumed by many statistical methods used for data analysis [16]. Normalization can also reduce systematic bias or technical variation and minimizes misidentification or overlooking of significant changes due to obscuration of smaller signals (as variance from more abundant metabolites tends to dominate the variance-covariance matrix) [16]. Beyond statistical analysis, other modules are available for use on MetaboAnalyst (such as Enrichment Analysis, Spectral Processing of LC-MS spectra, etc.) though they are not the focus of this study.

In the context of lipidomic research, MetaboAnalyst analyzes lipids exclusively at the molecular species level [18], as it considers lipids as any other metabolites. However, lipid molecules can be further considered by their building blocks of ‘head’ group and aliphatic ‘chains.’ By studying how lipid building blocks are altered, researchers can infer biochemical processes that have occurred (such as elongation, shortening, oxidation, reduction, etc.) [19]. The novelty of LipidOne stemmed from the need for a freely accessible, web-based tool that allows for closer analysis of lipid building blocks [19]. Developed in 2022, LipidOne has already received multiple updates, the most recent one of which expanded the tool’s support for animal model organisms, introduced additional color palette options, and added a lipid name conversion utility tool. Much like MetaboAnalyst, LipidOne supports both univariate and multivariate data analyses, offering techniques such as fold change analysis, *t*-test, volcano plots, ANOVA, PCA, and PLS-DA. Unique to LipidOne is the ability to apply statistical analysis techniques toward three lipidome levels: lipid class, lipid molecular species, and lipid building blocks.

Although there are several studies that review available tools for the analysis of ‘omics’ data, to the best of our knowledge, no review has directly compared MetaboAnalyst and LipidOne in the context of lipidomic data analysis. In this paper, we describe and compare the statistical analysis performance of MetaboAnalyst and LipidOne using a lipidomic dataset comparing control and post-optic nerve crush conditions in *Xenopus laevis* (African clawed frog) optic nerves; the dataset is publicly available on Metabolomics Workbench (ST002414) [20].

## 2. Materials and Methods

### 2.1. MetaboAnalyst 6.0

MetaboAnalyst is readily accessible online (https://www.metaboanalyst.ca/ (accessed on 26 August 2025)). The homepage provides an overview of the web tool, as well as the capabilities of the available modules. The lefthand section of the webpage contains hyperlinks for supplemental pages that contain examples of datasets accepted by the different modules, the MetaboAnalystR package if researchers are comfortable with using the R coding platform, tutorials, FAQs, a user forum, etc. The tool’s modules are accessed by pressing “Click here to start” at the center of the homepage. Within the Module Overview page, the module “Statistical Analysis [one factor]” was the module used and analyzed in this study.

### 2.2. LipidOne 2.3

LipidOne (https://lipidone.eu/ (accessed on 26 August 2025)) directs users to a homepage that provides examples of univariate and multivariate analysis figures that can be generated by the bioinformatic tool. Below this section contains recent publications that have used LipidOne, details on the tool’s most recent update, and hyperlinks to tutorials on the analytical functions available and how to prepare data for LipidOne. Pressing “Start Now” at the top of the homepage directs users to LipidOne’s statistical analysis module which is used and analyzed in this study.

### 2.3. Metabolomics Workbench

Metabolomics Workbench (https://www.metabolomicsworkbench.org/ (accessed on 24 July 2025)) is a public data repository developed by the National Institutes of Health’s (NIH) Data Repository and Coordinating Center (DRCC) that contains metabolomic metadata and experimental data spanning various species from multiple institutions [21]. The dataset used for this study was uploaded under the Study ID ST002414, titled “Mass spectrometry dataset of LC-MS Lipidomics Analysis of Xenopus Laevis Optic Nerve” (DOI: 10.21228/M8TM6T).

### 2.4. Methods

To provide a detailed comparison between MetaboAnalyst and LipidOne, a general workflow for lipidomic statistical analysis was performed using both tools: data upload, data integrity check and filtration, data normalization, and statistical analysis.

MetaboAnalyst allows researchers to upload datasets directly from Metabolomics Workbench using the “Statistical Analysis [one factor]” module, and our example study (ST002414) is readily accepted by MetaboAnalyst with 3432 lipids identified. LipidOne does not have an option to directly upload from Metabolomics Workbench. Instead, the tool requires a dataset in comma-separated (.csv) or tab-separated (.txt) format that follows specific rules. First, lipid nomenclature must comply with the “Molecular species level” of Lipidomic Standards Initiative (LSI) Guidelines [19], which standardizes practical shorthand notation of MS-derived lipid structures using common, officially accepted terms and LIPID MAPS terminology [22]. Another requirement is that there must be no duplicate lipid names within the dataset [19]. To fulfill these requirements, the dataset was again directly downloaded from Metabolomics Workbench and edited on Microsoft Excel. Identified lipids were converted into standardized nomenclature through A Reference List of Metabolite Names (RefMet) (https://www.metabolomicsworkbench.org/databases/refmet/index.php (accessed on 26 August 2025)), a tool that converts lipids into its corresponding LIPID MAPS ID. Due to the discrepancy in lipids identified by LipidSearch 5.0 (which was used by Study ST002414 to identify and quantify lipids) and lipids recognized by LIPID MAPS, certain lipids with no corresponding LIPID MAPS ID were left blank after conversion. Isomers of lipids were also converted into the same lipid name, resulting in multiple duplicates after conversion. Using Microsoft Excel, blanks were removed, and duplicates were consolidated into a single row (File S1). Though the dataset initially contained 3432 lipids identified using LipidSearch 5.0, removal of blank lipids post-conversion reduced the total to 2701 lipids, and consolidation of duplicates further reduced the total to 2546 lipids. Group names were edited to redefine groups and allow samples collected at different time points to be compared. To ensure that the difference in analyses between MetaboAnalyst and LipidOne is not confounded by significantly differing datasets, the dataset that is accepted by LipidOne (after nomenclature conversion and exclusion of blanks and duplicates) is used for both tools.

Data processing was performed using the same parameters for both tools’ analyses. A low-quality filter was applied to reduce the number of variables and increase power [17], removing lipids that had missing values for >50% of samples within groups. A low-variance filter was applied to exclude lipids with near-constant values across experimental conditions. MetaboAnalyst applies a default variance filter (top 60% most variable features retained, determined by interquantile range) for datasets exceeding 1000 samples, and therefore the same variance filter was applied to LipidOne’s dataset, albeit manually through Microsoft Excel. Remaining missing values after low-quality and low-variance filters were replaced according to user-specified parameters (MetaboAnalyst) and tool default (LipidOne). On MetaboAnalyst, remaining missing values were replaced by limit of detection (LoD; 1/5th of the minimum positive value of each variable) using group-specific values. LipidOne replaced numeric values that were absent or had a value of zero with a number equal to 1/10th of the minimum positive value of the variable after data upload [19]. Samples of our dataset were then Median normalized, and features were normalized by log transformation (base 10). Data normalization is highly recommended in lipidomics analysis due to the dynamic range of lipids present in a dataset [14].

Our study focused on the comparison of *Xenopus laevis* frogs that underwent optic nerve crush (ONC) surgery and frogs in the Control group that did not undergo surgery. Specifically, we compared optic nerve samples 7 days post-crush to 27 days post-crush, and Control samples (no ONC) collected at 7 days and 27 days to better understand the temporal dynamics of lipid remodeling in the regenerative model compared to control. All statistical analyses were performed on either MetaboAnalyst or LipidOne. Volcano plots were generated to characterize upregulated and downregulated lipids in both groups, and to showcase univariate analysis capabilities. MetaboAnalyst does not have a built-in test for heteroscedasticity but allows users to choose between Student’s *t*-test (equal variances) or Welch’s *t*-test (unequal variance). Because variance homogeneity could not be assumed, Welch’s *t*-test was used. LipidOne applies a default *t*-test for two-group comparisons, though its variance assumption is not specified and there are no options for users to modify it. For MetaboAnalyst, statistical significance was defined as FDR-adjusted q-value <0.05 with fold-change thresholds of 2. For LipidOne, statistical significance was defined as raw *p*-value <0.05 with the same fold-change threshold, as multiple-comparison correction was not available for volcano plots. PCA was performed as an unsupervised multivariate analysis to visualize clustering of samples and variance structure, as well as to showcase multivariate analysis capabilities of both tools. Variance explained by each principal component (R^2^) was calculated, and score plots of the first two principal components were generated. Group separation was formally tested using Permutational Multivariate Analysis of Variance (PERMANOVA) when available. PCA does not impose grouping information unlike PLS-DA and therefore avoids the risk of overfitting as our sample sizes for groups are limited relative to the number of lipids. Effect sizes were reported as fold changes for univariate analyses and as R^2^ values for PCA analyses. To showcase lipid building block analyses unique to LipidOne, Chain Unsaturation and Chain Length analyses were performed. All statistical tests were two-tailed, and adjusted q-value thresholds were consistently applied when available to minimize false positives.

To evaluate the features of both tools, explicit criteria across processing, normalization, statistical analysis, multiple testing correction, and output functions were prepared and listed in Table 1 below.

## 3. Results

### 3.1. Data Integrity Check and Filtration

Data integrity checking involves replacement of missing values and identification and removal of outliers. In MetaboAnalyst, users begin with Data check. This section displays data processing information, including number of samples, features, and groups. The section also quantifies the number of features with constant or single values across samples (which are automatically removed), the number and percentage of missing values, and whether missing value patterns differ significantly between groups, as determined by a Kruskal–Wallis test. Our dataset had five lipid features automatically removed for having constant or single values across samples. It contained 112,680 missing values (52.8%), and a Kruskal–Wallis test showed a highly significant difference in missing value patterns between groups (*p* = 8.41 × 10^–11^).

In the Data filter section of MetaboAnalyst, there are options to apply low-quality, low-repeatability, low-variance, and/or low-abundance filters on user datasets. Filtration is strongly recommended for untargeted lipidomics datasets due to the large number of variables present [17] and involves removing variables that are unlikely to be of use when modeling the data [15]. The low-quality filter, which removes features with too many missing values or heavily contaminated samples based on blank samples, defaults to removing features with missing values for >50% of its samples. Users can also choose group-wise threshold for missing value exclusion if missing value patterns differ significantly between groups. The low-repeatability filter allows for removal of features with a high percentage of relative standard deviation (RSD = SD/mean) based on quality-control (QC) replicates, thereby removing features with low repeatability. The low-variance filter removes features that are near constant throughout all experiment conditions; such features are detected using various methods including standard deviation (SD), interquantile range (IQR), median absolute deviation (MAD), RSD, or non-parametric relative standard deviation (MAD/median). Lastly, the low-abundance filter uses either mean intensity value or median intensity value to detect and remove features with very small values. For variance and abundance filters, MetaboAnalyst has default parameters that follow empirical rules: 5% filtration if there are less than 250 total variables, 10% filtration if total number of variables is between 250 and 500, 25% filtration if total number of variables is between 500 and 1000, and 40% filtration if there are greater than 1000 total variables [15]. For our example dataset, a low-quality filter was applied to remove features with missing values in >50% of samples. A 40% variance filter using IQR was applied according to the number of lipids present in the study; low-repeatability filter was not applied due to the lack of QC samples, and low-abundance filter defaulted to 0% and was therefore not applied. A total of 27 lipids were removed after applying the low-quality filter using the group-wise threshold, and 1006 lipids were removed by the low-variance (interquartile range) filter, leaving 1508 lipids after processing.

After data filtration, the Missing value section allows users to determine how they would like to deal with the remaining missing values. There are various options for missing value estimation. The method of estimation depends on the type of statistical analysis being performed, with more advanced methods such as k-nearest neighbors based on similar features (KNN) available for multivariate analysis [15]. The tool also lists the number and percentage of missing values after filtration. For our dataset, 5620 missing values remained (12.2% of the data), and these missing values were replaced by limit of detection (LoD; 1/5th of the minimum positive value of each variable).

LipidOne does not have built-in Data check, Data filter, and Missing value sections after uploading the dataset. However, the developers of LipidOne suggest removing lipids that contain constant or only zero values throughout all samples or within an experimental group [18], which would achieve the same processing as the automated step in the Data check section, as well as the low-quality filter step in the Data filter section of MetaboAnalyst. LipidOne accomplishes missing value replacement automatically, and therefore with less flexibility compared to MetaboAnalyst. By default, missing values are replaced with a number equal to 1/10th of the minimum positive value of the variable after data upload [19]. For LipidOne’s data processing, all steps were performed manually in Microsoft Excel. Five features with constant or zero values throughout all samples were removed. Eighteen features were then removed due to the low-quality filter using group-wise threshold. Finally, 1002 features were then removed due to the low-variance filter threshold, leaving 1521 lipids after processing.

1508 lipids were shared between the two workflows, 13 of which were unique in the LipidOne dataset, for an overall Jaccard similarity of 0.991.

### 3.2. Data Normalization

MetaboAnalyst has three categories available for data normalization: sample normalization, data transformation, and data scaling [15]. Sample normalization is performed to make samples more comparable, while transformation and/or scaling are applied to make lipids more comparable in magnitude to each other [16]. Our MetaboAnalyst dataset was normalized by median, and features were transformed by log (base 10) in order for the lipid species to adopt roughly a “bell curve” distribution and align samples for comparison. No autoscaling was applied.

LipidOne does not have a built-in normalization, transformation, or autoscaling feature. These steps were instead completed manually in Microsoft Excel. Missing value replacement was also performed manually prior to normalization, as this reflects the workflow of MetaboAnalyst. Missing values were replaced with a number equal to 1/10th of the minimum positive value of the lipid across samples, replicating what LipidOne would automatically perform after data upload. Normalization was then achieved by dividing each feature’s concentration by the median concentration of its respective sample. The normalized values were then log-transformed (base 10). No autoscaling was applied. To prevent LipidOne from reinterpreting zero values in the normalized dataset as missing data, zeros were replaced with 1 × 10^−6^. When performing PCA, LipidOne imposes an automatic normalization by median [18]. To avoid repeating the normalization step, a separate dataset was used for PCA on LipidOne that only underwent data processing and transformation.

### 3.3. Statistical Analysis

**Figure 1:**
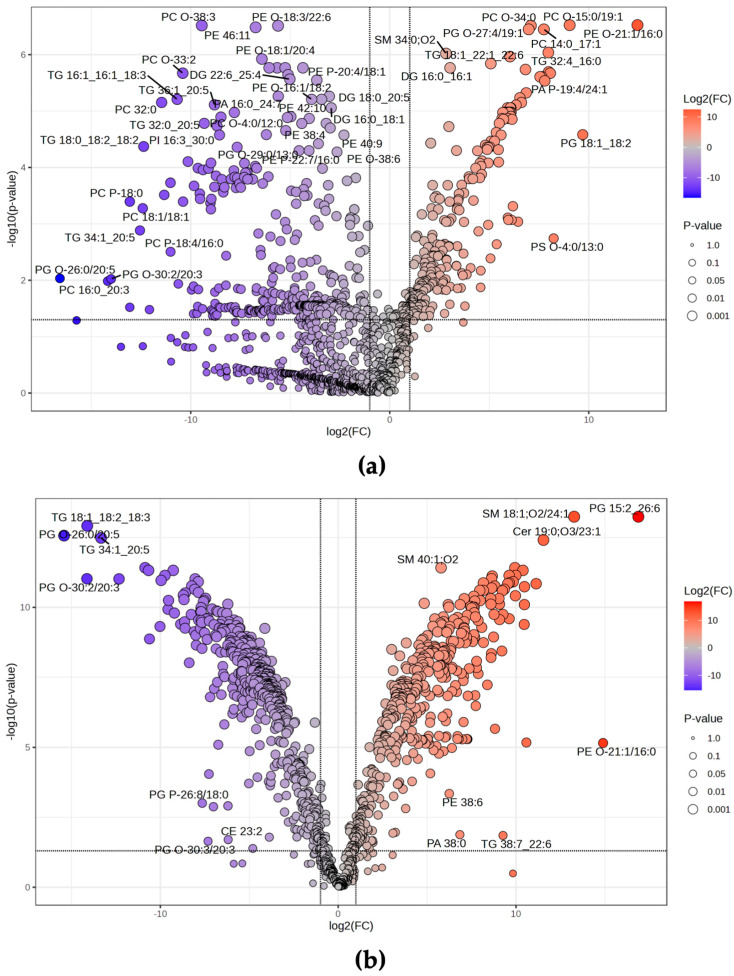
Volcano plots of significantly altered lipid species in *Xenopus laevis* optic nerve. Significant lipids between (**a**) Day 27 Crush vs. Day 7 Crush, and (**b**) Day 27 Control vs. Day 7 Control, generated using MetaboAnalyst. Significance is defined as FDR-adjusted q-value <0.05 and fold change threshold of 2. Red and blue dots signify upregulated and downregulated lipids, respectively.

MetaboAnalyst has a data editor section that allows users to exclude samples, groups, and/or features. In datasets with multiple groups, the data editor section is required to isolate two groups for comparison via volcano plot. Normalization steps were repeated every time the data was edited (normalization by median and log (base 10) transformation). Within the Volcano plot section of the tool, researchers can choose the direction of comparison, define *p*-value and fold change thresholds, choose if FDR adjustment is to be used for the *p*-value, choose between parametric or non-parametric testing, and choose between Student’s *t*-test (equal variance) or Welch’s *t*-test (unequal variance). Statistical significance was defined as FDR-adjusted q-value <0.05 and effect size as a fold change threshold of 2; direction of comparison was defined as Day 27 Crush/Day 7 Crush. A total of 225 lipids were found to be significantly upregulated, and 362 lipids were significantly downregulated (Figure 1a). In comparison, the Control group had a different distribution of significant lipid alterations, with 415 total lipids significantly upregulated and 474 lipids significantly downregulated (direction of comparison = Day 27 Control/Day 7 Control) (Figure 1b).

**Figure 2:**
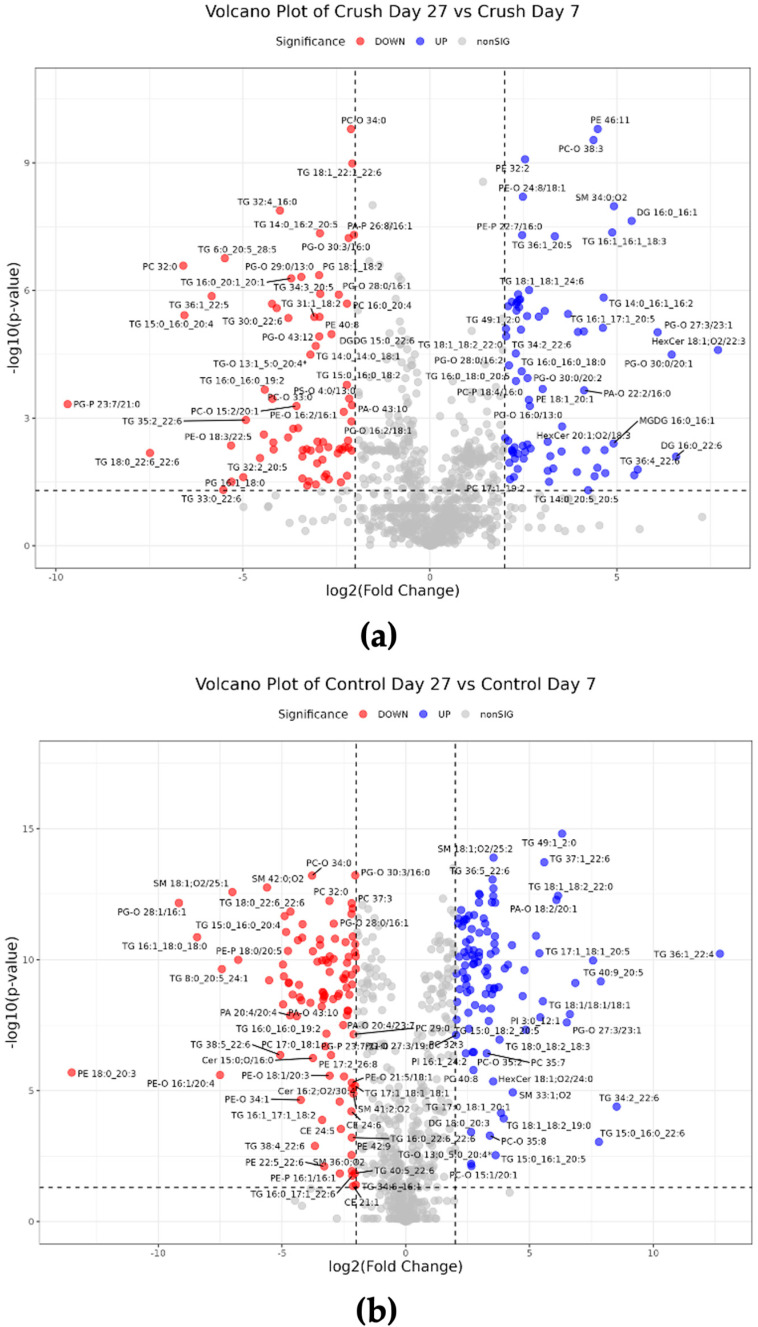
Volcano plots of significantly altered lipid species in *Xenopus laevis* optic nerve. Significant lipids between (**a**) Day 27 Crush vs. Day 7 Crush, and (**b**) Day 27 Control vs. Day 7 Control, generated using LipidOne. Significance is defined as raw *p*-value <0.05 and fold change threshold of 2. Blue and red dots signify upregulated and downregulated lipids, respectively. Lipids with an asterisk indicate that their annotations are compliant with RefMet standard nomenclature format but not present in the RefMet database.

LipidOne also offers various univariate analysis methods, though only volcano plots were generated in this study as a point of comparison. The same groups were compared: Day 7 Crush vs. Day 27 Crush, and Day 7 Control vs. Day 27 Control. When generating the volcano plot, researchers can define *p*-value and fold change thresholds on LipidOne, but no multiple-testing correction or variance assumption modification are offered. Therefore, statistical significance was defined as raw *p*-value <0.05, and effect size was defined as a fold change threshold of 2. Comparing Day 27 Crush to Day 7 Crush, 77 lipids were significantly upregulated, and 73 were significantly downregulated (Figure 2a). The amount and distribution of significant lipids differ to Day 27 Control vs. Day 7 Control, which found 105 significantly upregulated and 101 significantly downregulated lipids (Figure 2b).

Between Crush groups, 587 significant lipids were identified in MetaboAnalyst and 150 in LipidOne; 126 were shared between the analyses, while 611 were unique to one tool (Jaccard similarity = 0.21). More specifically, Jaccard similarity was 0.04 for upregulated lipids and 0.07 for downregulated lipids. For Control groups, MetaboAnalyst identified 889 significant lipids, while LipidOne identified 206 significant lipids; 144 were shared between the analyses, and 951 were unique to one tool (Jaccard similarity = 0.15). Jaccard similarity was 0.03 when comparing only upregulated or downregulated lipids.

**Figure 3:**
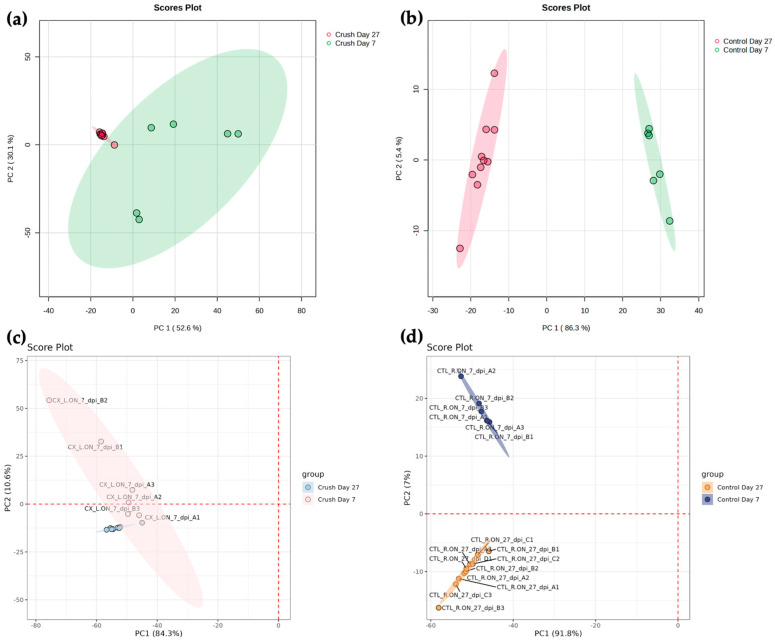
A comparison of principal component analysis (PCA) score plots of two programs. PCA score plots generated in MetaboAnalyst. Plots between (**a**) Day 27 Crush versus (vs) Day 7 Crush and (**b**) Day 27 Control vs. Day 7 Control. PCA score plots generated in LipidOne. Plots between (**c**) Day 27 Crush vs. Day 7 Crush and (**d**) Day 27 Control vs. Day 7 Control.

PCA of Crush groups revealed that PC1 and PC2 explained 52.6% and 30.1% of the total variance, respectively, whereas in the Control group, PC1 and PC2 explained 86.3% and 5.4% of the total variance, respectively. Score plots showed greater clustering of Day 27 Crush compared to Day 7 Crush, while Day 27 Control and Day 7 Control clustered distinctly from each other (Figure 3a,b). PERMANOVA confirmed that existing group labels explained a significant proportion of the variance seen in PCA (Crush groups: R^2^ = 0.48, *p* = 0.001; Control groups: R^2^ = 0.93, *p* = 0.001). Loading analyses were performed to identify lipids contributing most to variance for the principal components. For the Crush groups, separation on PC1 was associated with positive contributions from phosphatidylcholine (PC), phosphatidylglycerol (PG), and triglyceride (TG) species, while negative contributions were primarily from phosphatidylethanolamine (PE), cholesteryl ester (CE), sphingomyelin (SM), TG, PC, and PG species. Separation on PC2 was associated with positive contributions from SM, PC, PG, monoacylglycerol (MG), and phosphatidic acid (PA) species, and negative contributions from TG and PG species. For the Control groups, PC1 separation was associated with positive contributions from PG, TG, SM, and PA species, and negative contributions from PG, PE, SM, ceramide (Cer), TG, PC, and MG species. On PC2, positive contributions were mainly from CE, PG, PC, diacylglycerol (DG), and Cer species, as well as Coenzyme Q9, while negative contributions were primarily from PG, TG, and PE species.

PCA can also be performed in LipidOne. As mentioned previously, a separate dataset that only underwent processing and log (base 10) transformation was used due to LipidOne automatically applying a normalization by median when performing PCA. Performing the analysis on LipidOne allows users to perform scaling (Autoscaling, Pareto Scaling, or none) and choose which of five principal components to display on the *X*-axis and *Y*-axis. No scaling was performed, and PC1 was designated for the *X*-axis, with PC2 for the *Y*-axis. PCA of Crush groups revealed that PC1 and PC2 explained 84.3% and 10.6% of the total variance, respectively, whereas in the Control group, PC1 and PC2 explained 91.8% and 7.0% of the total variance, respectively. Day 27 Crush samples clustered more tightly than Day 7 Crush samples, while Day 27 Control and Day 7 Control samples were distinctly separated, primarily along PC2 (Figure 3c,d). Unlike MetaboAnalyst, LipidOne does not provide PERMANOVA analysis to test group separation. The tool generates a loading analysis graphic of top 20 loadings for PC1 and PC2 but does not provide a dataset listing the loading values of all lipids contributing to variance. It is also unclear whether lipids are ranked by the absolute or actual value of the loadings. Based on the top 20 loadings between Crush groups, separation on PC1 was associated with contributions predominantly from TG species, but also from PG, PA, SM, PE, and Cer species. PC2 separation was associated with contributions from PG, PC, CE, TG, and SM species. Meanwhile, top 20 loadings between Control groups show that PC1 separation was mainly attributed to TG species, with select PG, PC, PA, and DG species also contributing. PC2 separation was associated with PG, PE, SM, TG, Cer, MG, and HexCer species.

The top 20 loadings for PC1 and PC2 in the Crush and Control groups from LipidOne were compared with the corresponding top 20 positive loadings in MetaboAnalyst. For loadings in the Crush groups, no lipids were shared for PC1 and only 1 lipid was shared for PC2 (Jaccard similarity = 0.03). Similarly for Control groups, no lipids were shared for PC1 and only 3 lipids were shared for PC2 (Jaccard similarity = 0.08). Loadings from LipidOne were then compared with top 20 loadings ranked by absolute value in MetaboAnalyst. Comparison of loadings in Crush groups yielded the same results: no lipids were shared for PC1, and 1 lipid for PC2 (Jaccard similarity = 0.03). For Control groups, no lipids were shared for PC1, and only 1 lipid for PC2 (Jaccard similarity = 0.01).

Loading analysis results can be visualized in two different ways in MetaboAnalyst. The Loadings Plot tab can plot the loading distribution of lipids contributing to variance. Users can change principal components of the *X*- and *Y*-axis, and select either to label all lipids, only variables of interest (which is chosen by double-clicking the dot on the generated plot), or no lipids. Alternatively, loadings can be visualized using the Biplot tab, which also allows users to modify the principal components for the *X*- and *Y*-axis, and the number of top features to be displayed in the generated plot. In comparison, LipidOne can also generate a loading plot, but only principal components for the *X*- and *Y*-axis can be modified by users. Furthermore, only select lipids are labeled in the graphic by default.

Unique to LipidOne is the ability to analyze lipid classes and building blocks to gain more insight into the lipid changes occurring between groups of interest. The same univariate and multivariate analysis methods available for lipid species can be used for lipid class and lipid building block. Options for further analysis of lipid building blocks include but are not limited to Chain Length, Chain Unsaturation, Chain Profile, and Odd/Even Chain Ratio. Lipids can also be isolated by lipid class to allow for closer analysis of lipid species/building blocks belonging to only certain classes of interest. Note that although more readily accessible on LipidOne, the same isolation can be accomplished on MetaboAnalyst by excluding lipids outside of the desired lipid class using the Feature exclusion feature within the Data editor section. After exclusion however, statistical analysis is still only limited to lipid species. Though users can include only lipids belonging to a certain class for species analysis, comparison of groups at the class or building block level is not feasible.

**Figure 4:**
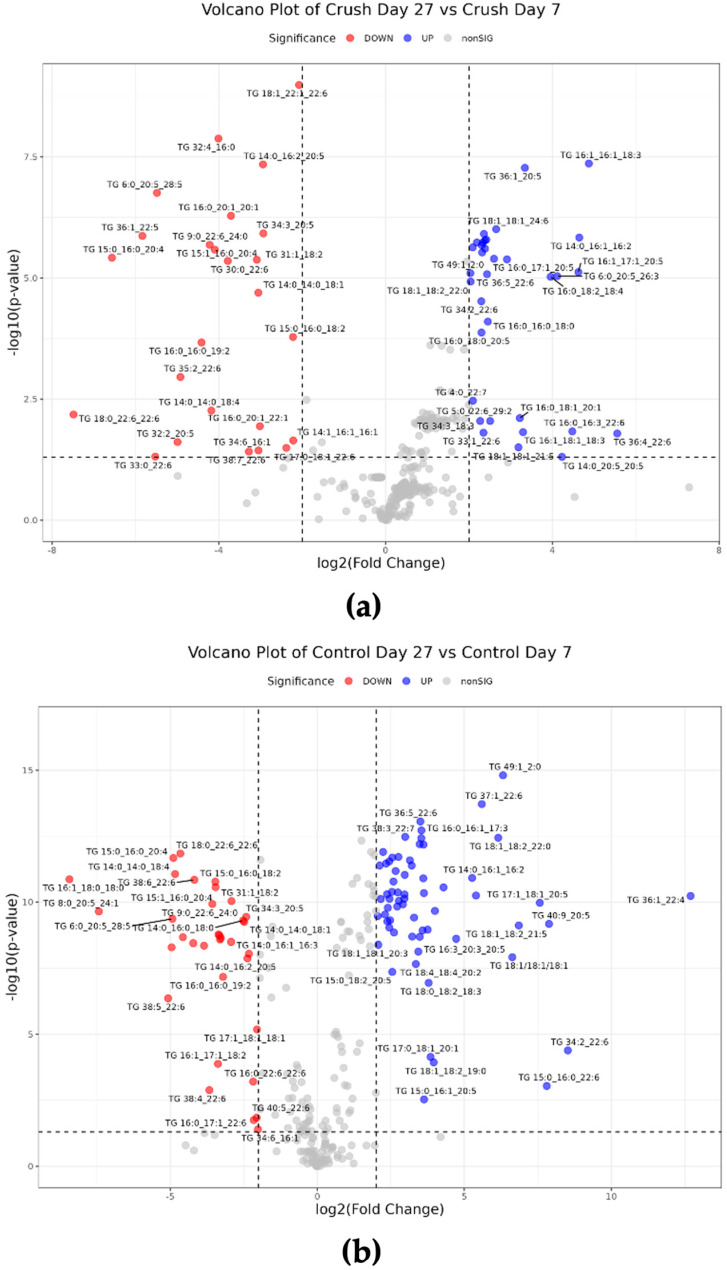
Volcano plots of significantly altered TG species in *Xenopus laevis* optic nerve. Significant lipids between (**a**) Day 27 Crush vs. Day 7 Crush, and (**b**) Day 27 Control vs. Day 7 Control, generated using LipidOne. Blue and red dots signify upregulated and downregulated lipids, respectively.

Indeed, analyzing the dataset using these statistical methods offered by LipidOne provided unique insights that were not obtained during statistical analysis using MetaboAnalyst. As mentioned previously, a study has shown that lipin1 elevates in mice after axotomy, which then triggers lipid metabolism to increase TG and decrease phospholipid production, ultimately limiting axon regrowth [7]. The PCA performed in LipidOne revealed that 12 of the top 20 loadings in PC1 between Crush groups were TG-class lipids. Similarly, TG-class lipids also comprised 15 of the top 20 loadings in PC1 between Control groups. To further classify the changes in TG, a volcano plot containing only TG lipids was generated, with statistical significance defined as raw *p*-value < 0.05 and biological significance as fold change threshold of 2. The TG-specific volcano plot revealed that 34 were significantly upregulated and 25 were significantly downregulated between Crush groups (Figure 4a). Between Control groups, 64 were significantly upregulated, and 34 were significantly downregulated (Figure 4b).

LipidOne’s building block analyses were applied to assess changes in chain unsaturation and chain length of TG species. These analyses use parametric *t*-tests (two-group comparisons) or ANOVA (multiple groups), although the software does not specify underlying assumptions such as equal variance. Significance was defined as FDR-adjusted q-value < 0.05. Based on Chain Unsaturation analysis, TG species with 7, 8, and 10 total double bonds significantly decreased in Crush by Day 27. Chain Length analysis revealed that TG with side chains containing 2, 13, 24, 40, and 49 carbons significantly decreased in Crush by Day 27, while TG with side chains containing 3, 5, 14, 29, and 30 carbons significantly increased by Day 27. Changes in TG unsaturation and chain lengths differed in the Control group. Chain Unsaturation analysis showed that TG with 0–5 and 7–9 double bonds significantly decreased by Day 27. TG with side chains containing 5, 9, 29, 30, 33, and 50 significantly increased by Day 27, while 25 other side chain lengths ranging from 2 to 49 carbons significantly decreased.

## 4. Discussion

MetaboAnalyst and LipidOne are both free web-based tools that make statistical analysis of lipidomic data accessible. They can both perform univariate and multivariate analyses to accommodate researchers’ experimental aims, but results show that there are key differences to consider when choosing either tool for lipidomic analysis.

MetaboAnalyst accepts more file types and dataset formats when uploading data for analysis. In the Upload section, the “Plain text file” option accepts .txt or .csv files with samples in rows or columns. Other options accept compressed files, mzTab 2.0-M files, XLSX files from Metabolon, and study ID directly from Metabolomics Workbench. In comparison, LipidOne only accepts .txt or .csv files with samples listed in columns.

Both tools offer in-tool data editing options. Specifically, MetaboAnalyst offers the ability to edit group names after uploading, and exclude samples, groups, or features through the Data Editor section. LipidOne offers less fine-tuned customization of the data. Group exclusion can be performed within the main statistical analysis page, sample exclusion through the Lipid Data Filtering feature within the Utility section of the tool, and a form of feature exclusion through inclusion/exclusion of samples according to lipid class.

LipidOne lacks data processing functions that are highly recommended when handling large datasets such as those generated in lipidomics research. Filtration of low-quality and low-variance lipids requires manual calculation using an outside resource such as Microsoft Excel. Despite choosing the same filtration thresholds, 13 lipids were uniquely retained in LipidOne’s data. Closer inspection of the R Command history available when using MetaboAnalyst reveals that another level of filtration is automatically applied along with the command to run the variance filter based on IQR. This added filter removes features that are not present in at least 20% of samples and is likely the source of discrepancy between the two datasets, despite having the same thresholds for low-quality and low-variance filters. Besides filtration, LipidOne lacks global data normalization, transformation, and autoscaling features, therefore requiring users to manually apply these prior to running most analyses in LipidOne. The exception covered in this study is PCA, which automatically applies normalization by median and allows users to choose between autoscaling, Pareto scaling, or no scaling prior to analysis. Missing value replacement in MetaboAnalyst is highly customizable, while LipidOne defaults to 1/10th of the minimum positive value of the variable. However, this was also manually performed prior to data upload, as normalization by median and log (base 10) transformation would have been skewed by missing values. In turn, zeros in the dataset after manual normalization and transformation had to be replaced by 1 *×* 10*^−^*^6^ to prevent LipidOne from labeling them as missing values. Another important distinction between the two tools is the ability to recalibrate normalization and data transformation upon exclusion of samples, features, or groups. When Crush or Control groups were isolated for analysis, no features were removed from consideration, therefore median values for normalization were not affected. However, if users exclude features from analysis (such as to isolate lipids belonging only to certain lipid classes), its effect on data normalization and transformation should be considered. LipidOne does not offer normalization within the tool, thus normalization is not calibrated to the median of only the lipids being considered whenever features are excluded (such as the lipid building block and TG/PL-only volcano plot analyses performed in this study).

LipidOne does not consistently offer corrections for multiple comparison testing which contributed to differences in the quantity of significant lipids in the volcano plot analyses (due to different definitions of statistical significance). FDR correction is offered for dedicated *t*-test/ANOVA analyses such as for lipid building block analyses of chain length and unsaturation. Both programs may benefit from offering options for softer corrections, such as sequential goodness of fit, as lipids are usually not all truly independent (with one lipid possibly represented by several ions or adducts) and can be significantly overcorrected [11].

MetaboAnalyst contains a dedicated section to show the R command history, which allows users to inspect what specific commands the tool runs during processing, normalization, statistical analysis, etc. This feature allowed an automatically added feature filter to be recognized while running the low-variance filter based on IQR. LipidOne, which is a stand-alone software program written in Perl language [19], does not have a dedicated section for its command history, therefore providing less transparency regarding its workflow.

In this study, volcano plot analysis was used as an example of univariate analysis capabilities. MetaboAnalyst offers robust statistical and visual customization options for users. Statistically, users can customize *p*-value and fold change thresholds, opt for FDR correction, and label the analysis as parametric/non-parametric and group variance as equal/unequal. A .csv file can be downloaded which contains the significant lipids and their *p*-value or FDR-corrected q-value, fold change, log2(fold change), and log2(*p*- or q-value). Visually, users can choose to label only a certain number of top features or all significant features, and what image format, resolution, and size to generate. In comparison, LipidOne does not offer FDR correction or options to label the test as parametric/non-parametric or having equal/unequal variance. A .csv file can be downloaded which lists significant lipids and their log2(*p*-value), log2(fold change), and significance (listed as either UP or DOWN). Visually, users are unable to edit the color designations for upregulated and downregulated lipids, and only a .png file can be downloaded. The size and resolution of the file cannot be edited. A substantial discrepancy was found between the two tools’ volcano plot results, which can be attributed to steps that altered the dataset after data processing, such as normalization, transformation, and missing value replacement. The different definitions of statistical significance between the two tools due to a lack of FDR correction in LipidOne also contributed to this difference. Furthermore, the type of *t*-test run by the tools may be different, but this is speculative due to unclear assumptions in LipidOne’s *t*-test.

PCA was used to compare multivariate capabilities between the two tools. MetaboAnalyst provides more options for data visualization, including an overview plot, biplot, and synchronized 3D plots which are not available in LipidOne. Statistically, MetaboAnalyst provides PERMANOVA results which allows confirmation of visually observed separation between groups in the score plot. Users can also download a .csv file containing loading values of lipids for multiple principal components. Visually, users can choose group colors for the score plot and choose what image format, resolution, and size to generate. Meanwhile, LipidOne’s PCA is statistically limited by its lack of PERMANOVA testing. It is also limited to only score plot and loading plot for visualization. Loading values are not provided; only the top 20 loadings are listed and for only the two designated principal components being observed. Unique to the tool is the ability to visualize potential sample outliers through an outlier plot graphed based on Mahalanobis distance. Visually, users can edit group colors like in MetaboAnalyst, but no customization of image format, resolution, or size is provided. Furthermore, color designation of lipid classes and the number of labeled features for the loading plot cannot be customized. A substantial discrepancy was also found between the two tools’ PCA results and top 20 loadings. Like in univariate analyses, differences observed can be attributed to steps that altered the dataset after processing. The definition of top 20 loadings in LipidOne is also unclear (top 20 positive loadings or top 20 loadings ranked by absolute value); nonetheless, comparison to the top 20 loadings in MetaboAnalyst ranked by positive and absolute values both provided substantial differences, pointing to key differences in how the dataset was altered after processing and/or how the PCA was performed.

MetaboAnalyst lacks the ability to perform statistical analysis on lipid classes and lipid building blocks, a gap that served as the purpose for LipidOne’s inception [19]. Studying these aspects of lipidomics can offer unique insights into lipid changes between groups, as demonstrated in this study through further characterization of TG unsaturation and chain lengths in Crush and Control groups. Though there were multiple TG species that were significantly upregulated and downregulated by 27 days post-ONC, unsaturation analysis showed that TG with higher degrees of unsaturation were significantly depleted, suggesting selective mobilization of polyunsaturated fatty acids (PUFA) from PUFA-rich TGs. Because PUFAs serve as essential precursors for phospholipid remodeling and bioactive lipid mediators [18,23], this selective depletion could suggest regeneration-oriented lipid metabolism. Given LipidOne’s current statistical limitations, particularly regarding *t*-tests assumptions, these findings should be interpreted with caution. Furthermore, this topic lies beyond the scope of this study and is better suited for further investigation in future experiments. Though not demonstrated in this study, LipidOne may also possibly facilitate the study of a sample’s lipidomic plasticity and flexibility, two proposed properties that may be inherent in an organ or bodily fluid [24]. Lipid plasticity is defined as the ability of a lipid class to undergo changes in its lipid species’ composition through alterations in fatty acid length, unsaturation, or abundance in response to an experimental condition [24], while lipid flexibility is the ability of a lipidome to undergo changes in response to different conditions, measured as the magnitude of changes on various levels of a lipidome [24,25]. Lipid plasticity was estimated as the number of double bonds and the weighted mean of the number of carbon atoms in the hydrocarbon moiety of a lipid class [24]. Both lipid plasticity and flexibility have various experimental implications. The study by Surma et al. regarding the lipidome of different mice organs found organ-specific differences as the largest cause of variation in data, with brain lipid classes showing very low lipid plasticity compared to other organs. Therefore, significant alterations in lipids may be more meaningful in the brain or other organs with low lipid plasticity compared to organs with high lipid plasticity [24]. Meanwhile, organs with innately high lipid flexibility may require fewer biological replicates in an experiment, as organs with high lipid flexibility would show larger alterations in response to a given condition [24]. In the context of axon regeneration, studying lipid plasticity and flexibility may prove useful when comparing different model organisms or cell types (CNS vs. PNS neurons), and LipidOne may be an appropriate tool for such a study.

A key limitation of our study is the reliance on a single lipidomic dataset, which may risk overfitting conclusions to its specific structure. Though our findings provide strong initial insights into both tools’ capabilities, further validation using multiple independent datasets is required to demonstrate generalizability and further strengthen our claims.

Overall, both tools offer statistical analysis options that are useful in lipidomics. MetaboAnalyst outperforms LipidOne regarding data upload, data processing, normalization, multiple comparison testing correction, workflow transparency, and statistical and visual customization of univariate and multivariate analyses. LipidOne uniquely offers analysis of lipidomic data beyond the molecular species level. However, the tool must improve the amount of customization available for statistical analysis, particularly variance assumptions for univariate analyses and PERMANOVA testing for PCA. It would also be beneficial for command history and .csv files of multivariate results to be readily available for increased transparency and reproducibility when utilizing the tool. Combined use of the tool is possible since MetaboAnalyst allows users to download processed and normalized data in .csv format. Theoretically, users can process, normalize, and analyze lipidomic data on MetaboAnalyst, then download the normalized data for further lipid class/building block analysis on LipidOne. Table 2 summarizes the results of the evaluation criteria created to compare the two tools.

## Figures and Tables

**Table 1:** Criteria and definitions for comparison of features between MetaboAnalyst and LipidOne.

	Explicit Criterion
Data upload	Does the tool support multiple file types and dataset formats?
Data processing	Does the tool allow user-defined filters for feature exclusion?
Missing value replacement	Does the tool allow replacement of missing values?
Data normalization	Does the tool allow user-defined normalization?
Data transformation	Does the tool allow user-defined data transformation?
Data scaling	Does the tool allow user-defined data scaling?
Class-level statistics	Can statistical tests be run directly on lipid classes?
Building block-level statistics	Does the tool support analysis of lipid building blocks (such as chain length, unsaturation, odd/even chains)?
Univariate statistics	Does the tool support commonly used univariate analyses?
Multivariate statistics	Does the tool support commonly used multivariate analyses?
Multiple testing correction	Is multiple testing correction (such as FDR) available and documented?
Reproducibility support	Is a script or command history available for users to inspect and replicate the exact processing and statistical analyses performed by the tool?
Visualization options	Are there customizable options for images generated? Are there options to edit image file type, resolution, and size?
Export formats	Can results be exported in .csv format? Can datasets be downloaded after changes are made in the tool (such as normalization or scaling)?

**Table 2:** Results of evaluation criteria comparing features of MetaboAnalyst and LipidOne.

	MetaboAnalyst 6.0	LipidOne 2.3
Data upload	Accepts .txt, .csv., compressed, mzTab, XLSX from Metabolon, or directly from Metabolomics Workbench; accepts samples in rows or columns	Accepts .txt and .csv files; accepts samples in columns
Data processing	Low-quality, low-variance, and low-repeatability filters are user-defined	None
Missing value replacement	User-defined	Automatic replacement of zero/missing values with 1/10th of lowest value of variable
Data normalization	User-defined and recalibrated after every feature, sample, and/or group exclusion	Normalization by median automatically performed for PCA
Data transformation	User-defined and recalibrated after every feature, sample, and/or group exclusion	None
Data scaling	User-defined and recalibrated after every feature, sample, and/or group exclusion	Autoscaling or Pareto scaling options available for select analyses
Class-level statistics	None	Univariate and multivariate analyses
Building block-level statistics	None	Univariate analyses
Univariate statistics	Fold change, *t*-test, volcano plot, ANOVA, correlation heatmaps, pattern search, correlation networks	Bar graph, *t*-test, ANOVA, correlation, volcano plot, biomarker discovery, box plot, ROC analysis, sequence analysis
Multivariate statistics	PCA, PLS-DA, sPLS-DA, orthoPLS-DA	PCA, PLS-DA, sPLS-DA, orthoPLS-DA, ROC analysis
Multiple testing correction	FDR correction	FDR correction only for select analyses (unavailable for volcano plot)
Reproducibility support	R Command history available	None
Visualization options	Customization for group colors, significance colors, and feature labels; User-defined image file type, resolution, and size for download	Customization for group colors; only .png file can be downloaded and without specification of resolution or size
Export formats	Results can be downloaded in .csv format; datasets can be exported in .csv format	Only select results can be downloaded in .csv format; no option to download datasets

## Data Availability

The original data presented in the study are openly available in Metabolomics Workbench at DOI: 10.21228/M8TM6T.

## Supplementary Materials

The following supporting information can be downloaded at: https://www.mdpi.com/article/10.3390/mps8050110/s1, File S1: manuscript-supplementary.xlsx.

## Abbreviations

The following abbreviations are used in this manuscript:

PC	Phosphatidylcholine
CNS	Central nervous system
PL	Phospholipid
LC/MS	Liquid chromatography coupled with mass spectrometry
ANOVA	Analysis of variance
PCA	Principal component analysis
PLS-DA	Partial least squares discriminant analysis
FDR	False discovery rate
NMR	Nuclear magnetic resonance spectroscopy
GC	Gas chromatography
FAQs	Frequently asked questions
NIH	National Institutes of Health
DRCC	Data Repository and Coordinating Center
.txt	Tab-delimited text file
.csv	Comma-separated values file
LSI	Lipidomic Standards Initiative
RefMet	A Reference List of Metabolite Names
ng/mL	Nanograms per milliliter
KNN	K-nearest neighbors
LoD	Limit of detection
RSD	Relative standard deviation
SD	Standard deviation
QC	Quality control
IQR	Interquantile range
MAD	Median absolute deviation
ONC	Optic nerve crush
PERMANOVA	Permutational Multivariate Analysis of Variance
R^2^	Proportion of variance explained by each principal component
PE	Phosphatidylethanolamine
PG	Phosphatidylglycerol
CE	Cholesteryl ester
TG	Triglyceride
HexCer	Hexosylceramide
SM	Sphingomyelin
Cer	Ceramide
LSM	Lysosphingomyelin
DG	Diacylglycerol
LPC	Lysophosphatidylcholine
PA	Phosphatidic acid
PC-O	Phosphatidylcholine with an alkyl ether-linked fatty acid
PC-P	Phosphatidylcholine with an alkenyl ether-linked fatty acid
PE-O	Phosphatidylethanolamine with an alkyl ether-linked fatty acid
PE-P	Phosphatidylethanolamine with an alkenyl ether-linked fatty acid
